# Graves’ disease thyroid dermopathy: a case report

**DOI:** 10.1186/s13256-024-04462-x

**Published:** 2024-04-07

**Authors:** Loay Tashkandi, Afaf Alsagheir, Saud Alobaida, Raghad Alhuthil

**Affiliations:** 1 King Faisal Specialist Hospital and Research Centre, 11211 Riyadh, Saudi Arabia; 2 Department of Pediatrics, King Faisal Specialist Hospital and Research Centre, Al Takhassousi & 12713, 11211 Riyadh, Saudi Arabia; 3 Department of Dermatology, King Faisal Specialist Hospital and Research Centre, 11211 Riyadh, Saudi Arabia

**Keywords:** Graves’ disease, Dermopathy, Myxedema, Koebner phenomenon, Case report

## Abstract

**Background:**

Graves’ disease is the autoimmune activation of the thyroid gland causing diffuse enlargement and hyperfunction of the gland. Manifestations of Graves’ disease are multisystemic and include thyroid orbitopathy; pretibial myxedema, also referred to as thyroid dermopathy; and thyroid acropachy, described as a severe form of thyroid dermopathy. Our paper focuses on an atypical case of thyroid dermopathy.

**Case presentation:**

An 11-year-old Saudi male presented with a prominent diffuse goiter and exophthalmos. Investigations were consistent with a diagnosis of Graves’ disease. The physical exam showed diffuse, non-pitting swelling of the ankle and penis, mimicking a lymphatic malformation. Further, multiple nodules were found on the hands and feet. Treatment of the nodules with cautery resulted in more severe nodules.

**Conclusion:**

This report describes rare presentations of thyroid dermopathy mimicking lymphatic malformation. The Koebner phenomenon can explain this patient’s atypical presentations. Intralesional injections of triamcinolone and total thyroidectomy showed clear improvement.

## Introduction

Graves’ disease (GD) is the most common cause of hyperthyroidism in childhood. It is defined as the autoimmune activation of the thyroid gland causing diffuse enlargement and hyperfunction of the gland. The autoantibodies implicated in the pathogenesis of the disease interact with the thyroid-stimulating hormone (TSH) receptor in the thyroid follicles; these include anti-thyroid peroxidase antibodies (anti-TPO), thyroglobulin antibodies (anti-thyroglobulin), and TSH-binding inhibitory immunoglobulins. The autoantibodies block the interaction of TSH with its receptor and mimic its function [1].

The manifestations of GD are multisystemic, including thyroid orbitopathy (TO); pretibial myxedema, also referred to as thyroid dermopathy (TD); and thyroid acropachy (TA), described as a severe form of TD [2, 3].

Thyroid dermopathy is an uncommon manifestation of GD; the onset usually ranges from 12 to 24 months after diagnosis. Its incidence in patients with GD ranged between 0.5% and 4.4% [4]. Furthermore, an association between the severity of TO and the prevalence of TD was made clear; among severe TO patients requiring aggressive intervention, 13% also had TD [1].

Thyroid dermopathy is described as bilateral, asymmetric, non-pitting thickening of the skin that may present in different forms. A retrospective study of 178 patients described the incidence of each form: isolated nonpitting edema (43.3%), well-demarcated nodules or plaques (27.0%), and more severe cases presented with elephantiasis (2.8%). The nodules may be hyperpigmented or violaceous. The lesions may have an orange-peel appearance. Furthermore, the lesions can be asymptomatic, pruritic, or painful [5].

This highlights the need for increased reporting of such extra-thyroid manifestations in the pediatric population, as discussed in this case report.

## Case presentation

An 11-year-old Saudi male was referred to our hospital from a local hospital in Haʼil (north-western Saudi Arabia) with thyrotoxicosis and post-trauma penile swelling for further management. He presented to our hospital in July 2018 with a history of hyperthyroidism and thyroid goiter with diffuse toxic goiter for 1 month.

A pediatric multidisciplinary team managed the patient, including endocrinologists, urologists, dermatologists, ophthalmologists, and otolaryngologists. At presentation, he had prominent diffuse goiter, exophthalmos with no tremors, and pretibial myxedema for 1.5 months. History showed an increase in appetite and weight loss. However, no palpitation, excessive sweating, or tremors were present. Patient was already started on oral methimazole 5 mg, twice daily, for 2 months, with good compliance, and no other past medical history. Family history noted that the parents are third-degree consanguineous; he is enrolled in school and progressing as expected for his age.

Physical examination noted the patient looking well, with a heart rate of 100 beats/minute, a regular rhythm, a weight of 29 kg (10th percentile), and a height of 140 cm (50th percentile). Respiratory examination showed equal bilateral air entry, with no added sounds. Cardiovascular examination was normal: S1 and S2 heart sounds heard with no added sounds. Abdomen was soft and not distended, there was no tenderness, and no masses were palpated. Neurological examination was unremarkable.

Initial investigations were consistent with a diagnosis of Graves’ disease; white blood cell count (WBC) was 5.63/L $\times {10}^{9}$ (4.5–13.0 $\times {10}^{9}$/L), hemoglobin was 11.3 g/dL (12.0–15.2 g/dL), hematocrit was 38.8% (36–47%), mean corpuscular volume (MCV) was 74.9 fL (78–96 fL), and mean corpuscular hemoglobin (MCH) was 21.8 pg (25–35 pg). Sodium was 139 mEq/L (136–145 mEq/L), chloride was 104 mEq/L (95–105 mEq/L), and calcium was 2.46 mmol/L (2.2–2.7 mmol/L). Regarding liver function tests, total bilirubin was 8.5 μmol/L (5–17 μmol/L), direct bilirubin was 2.3 μmol/L (1.7–5 μmol/L), AST was 20.9 units/L (15–46 units/L), ALT was 14.4 units/L (8–36 units/L), ALP was 359 units/L (85–400 units/L), and albumin was 40.8 g/L (34–54 g/L). Renal function tests found creatinine of 33 units/L (10–90 units/L), and urea was 2.6 mg/dL (5–20 mg/dL). Thyroid-stimulating hormone (TSH) was 0.005 mU/L (0.27–4.2 mU/L), total triiodothyronine (T3) was 8.9 nmol/L (1.3–3.1 nmol/L), and free thyroxine (FT4) was 62.11 pmol/L (12–22 pmol/L). The thyroid ultrasound noted mild diffuse enlargement of both lobes and isthmus, heterogeneous echo pattern, and no focal lesions—diffuse mild goiter. The diagnostic iodine uptake scan showed a mildly enlarged thyroid gland with homogeneous increased radiotracer uptake. The 4-hour I-123 thyroid scan uptake was 73.5%, and 24-hour was 63%. The thyroid antibodies were tested. Anti-thyroglobulin was 144 U/mL (< 115 U/mL), while anti-TPO was 413 U/mL (< 34 U/L); thyroid-stimulating hormone (TSAB) was 575% (< 140%). Thus, the patient was continued on methimazole and was seen regularly at a follow-up clinic.

In June 2019, the patient was referred to the ophthalmology clinic owing to progressive exophthalmos, and the visual screening showed 20/30 vision for both the left and right eye. Ophthalmology management was conservative with regular follow-up.

**Fig. 1:**
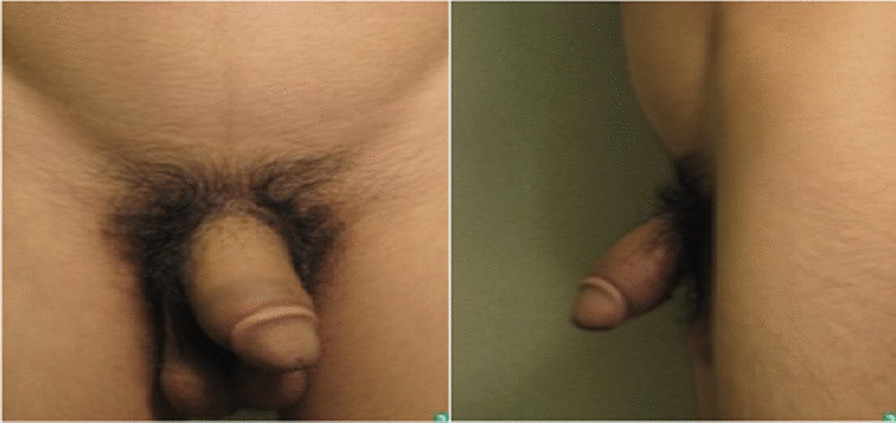
Anterior and lateral views showing diffuse penile swelling

**Fig. 2:**
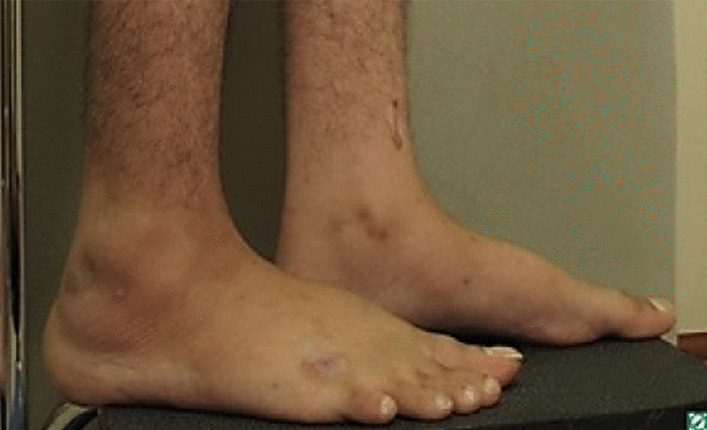
Lateral view showing diffuse ankle swelling

Furthermore, the patient noticed bilateral soft tissue swelling of the feet and penile region, with nodules on the hands and feet, and was referred to dermatology in August 2019. On physical examination of the ankle and penis, the swelling was diffuse, tense, and non-pitting (Figs. 1 and 2). The swelling on both ankles and in the penile region raised the possibility of an associated vascular malformation.

**Fig. 3:**
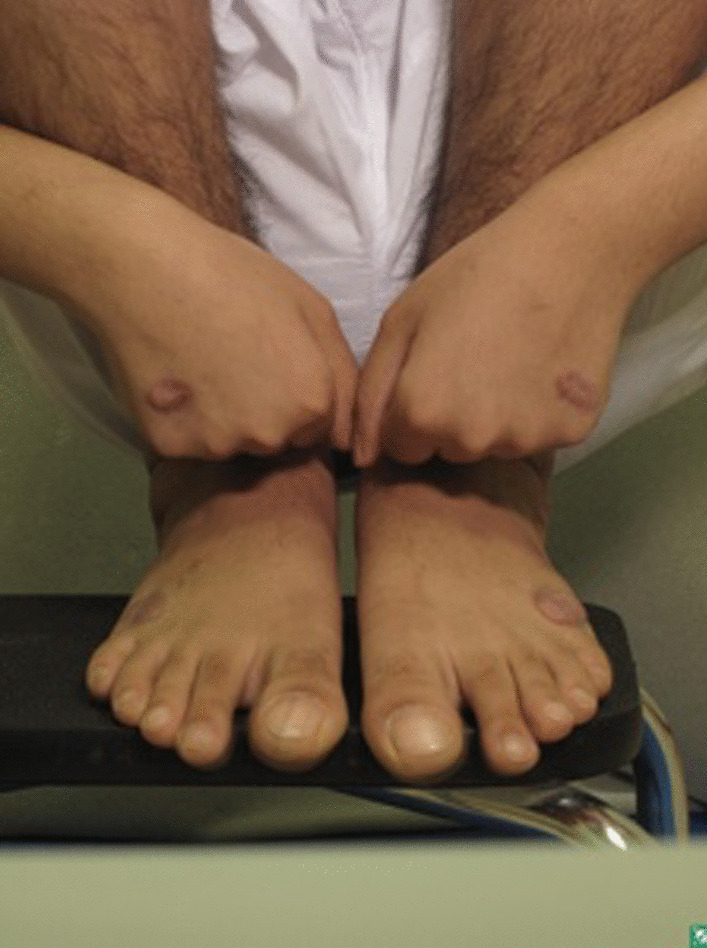
Multiple nodules on sites of cautery

Detailed physical examination noted deep swelling imitating lymphatic malformation with multiple nodules; right lateral foot near lateral malleolus (9 × 7.5 cm), fifth suitable toe base (3 × 1 × 5 cm), right foot medial aspect (1 × 1 cm), left lateral foot near lateral malleolus (8 × 7 cm), dorsum of left foot (3 × 2 cm), left fifth toe base (3 × 2 cm), and a solitary lesion on both the right and left hands were observed. On both hands and feet, lesions were treated with cautery initially. Upon follow-up in November 2020, new, more severe, brownish nodules appeared on the areas treated (Fig. 3).

**Fig. 4:**
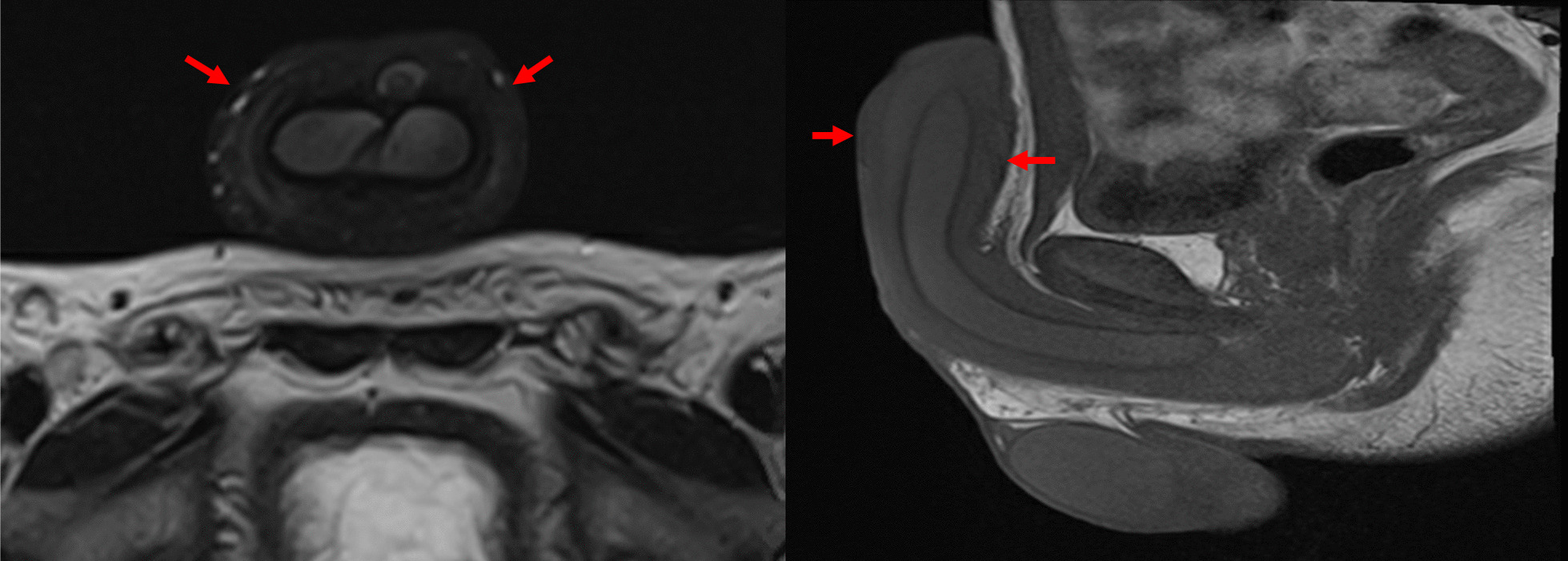
Pelvis magnetic resonance imaging showed non-specific subcutaneous soft tissue thickening surrounding the penis (see the red arrows). No evidence of collections/lymphatic malformation within the pelvic region

**Fig. 5:**
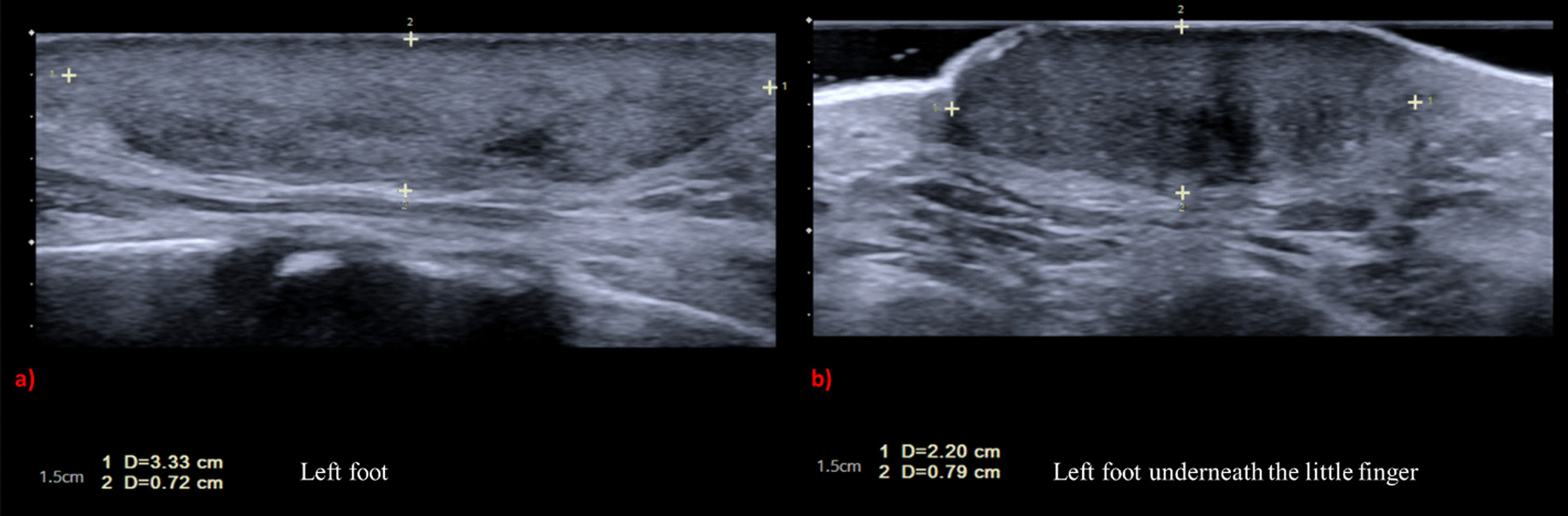
Standard ultrasound scan of the feet showed bilateral multiple fairly well-defined heterogeneously hypoechoic cutaneous/subcutaneous nodules. The largest nodule is seen around the left ankle joint, which measures 3.33 × 0.72 cm (**a**). The similar smaller lesion seen in the dorsum of the left measures 2.20 × 0.79 cm, with no evidence of extension to deep structures and no internal vascularity or calcifications (**b**). These nodules are of non-specific sonographic appearance

**Fig. 6:**
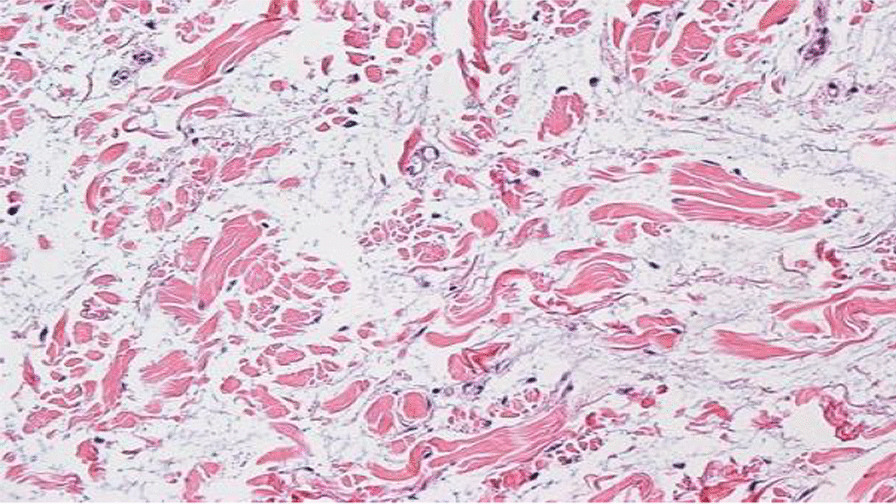
Mucin accumulation in the dermis

Workup for the patient started with magnetic resonance imaging (MRI) of the penile region and showed subcutaneous soft tissue swelling around the penis, with no focal nodules or masses, no loculated fluid collections, and no vascular malformations (Fig. 4). Furthermore, ultrasound imaging of the feet was done, which reported left foot edema and bilateral, multiple, well-defined, heterogeneously hypoechoic cutaneous and subcutaneous nodules (Fig. 5). A 4 mm skin biopsy was taken from the right foot and showed mucin accumulation in the dermis, consistent with pretibial myxedema (Figs. 3, 6). Management of the lesions with multiple rounds of intralesional injection of triamcinolone showed clinical improvement.

The patient had blepharospasm and severe thyroid orbitopathy that required orbital decompression and bilateral lateral tarsorrhaphy in March 2021 owing to progressive worsening despite medical management. Eventually, the patient underwent total thyroidectomy in June 2021; he showed further clear improvement in TD, and was prescribed daily oral levothyroxine 100 mcg.

The patient’s last follow-up was in the endocrine clinic (May 2023). He was compliant with the levothyroxine, his TD and TO had improved, and he was considered clinically euthyroid. His FT4 was normal (14.1 pmol/L), and TSH was high (16.200 mU/L). He had no active complaints, and his parents were satisfied with his overall improvement.

## Discussion

The patient, an 11-year-old Saudi male, was referred to the hospital with thyrotoxicosis and penile swelling post-trauma. Investigations were consistent with a diagnosis of Graves’ disease. A multidisciplinary team managed his condition, which included prominent diffuse goiter, progressive exophthalmos, and pretibial myxedema. The patient had a unique presentation with soft tissue swelling of the feet and penile region, and nodules on the hands and feet that became more severe with cautery treatment, but he improved with intralesional injections of triamcinolone. Eventually, the patient underwent orbital decompression, blepharospasm, and finally total thyroidectomy showing clear clinical improvement.

The Koebner phenomenon was first described in 1878; it is the appearance of new skin lesions subsequent to trauma on areas previously unaffected. In that regard, the patient is described as Koebner-positive [6]. This phenomenon explains the atypical presentation of the patient’s thyroid dermopathy, which was seen as a lymphatic malformation imitator present on the lower limbs bilaterally and the penile shaft, with development of new lesions following trauma to the sites. This position is further strengthened upon the resolution of the lesions only after total thyroidectomy.

A substantial amount of research has been directed at TO compared with TD. It has been speculated that both presentations share similar pathogenesis where cellular, immunologic, molecular, and mechanical factors result in glycosaminoglycan accumulation, causing expansion of the connective tissue and leading to lymphedema through obstruction of the lymphatic microcirculation [1].

Research into the prevention of TD needs to be improved. However, with the assumption of shared pathogenesis between TD and TO, one may be able to prevent TD in the same manner as TO. Normalization of thyroid function is essential in the management and prevention of TO [1].

As for the treatment of TD, standard therapy has been topical corticosteroids. Furthermore, intralesional injections would be the preferred treatment for plaques and nodules. It has been reported that surgical excision can improve short-term outcomes of severe cases if used in conjunction with octreotide. Lastly, possible directed therapy options include plasmapheresis, octreotide, tumor necrosis factor (TNF) inhibitors, tyrosine kinase inhibitors, rituximab, tocilizumab, TSH-receptor‐blocking antibodies, and IGF‐1-receptor‐blocking agents [1].

In this case, the presentation is unique and resembles a lymphatic malformation involving both feet and the penile region; to our knowledge, a similar presentation has not been described in the past. Given that an intralesional form was not injected in the penile region owing to the safety profile, no improvement was seen.

This case shows pretibial myxedema that developed Koebner’s phenomenon on the sites of cautery, where more severe nodules developed.

The long-term outcomes of these rare presentations need to be followed. Thus, monitoring and publishing long-term outcomes of the proposed management options should be a topic for future studies.

## Conclusion

In summary, we present a case with a rare presentation of thyroid dermopathy mimicking lymphatic malformation. The Koebner phenomenon can explain this patient’s atypical presentation. The rarity of the presentations has been emphasized by the lack of studies on the topic, specifically in the pediatric population. The severe manifestation of thyroid dermopathy responded well after intralesional triamcinolone and total thyroidectomy. Therefore, we recommend that management of such atypical cases of TD should consist of normalization of the thyroid function and intralesional injections.

## Data Availability

The datasets used in the current study are available from the corresponding author upon reasonable request.

## References

[1] Fatourechi V (2012). Thyroid dermopathy and acropachy. Best Pract Res Clin Endocrinol Metab.

[2] Kraus CN, Sodha P, Vaidyanathan P, Kirkorian AY (2018). Thyroid dermopathy and acropachy in pediatric patients. Pediatric Dermatol.

[3] Sanchez DP, Sonthalia S. Koebner phenomenon. U.S. National Library of Medicine; 2023. https://pubmed.ncbi.nlm.nih.gov/31971748/#:~:text=The%20Koebner%20phenomenon%20(KP)%2C,unaffected%20skin%20secondary%20to%20trauma. Accessed 10 May 2023.

[4] Fatourechi V (2005). Pretibial myxedema. Am J Clin Dermatol.

[5] Malabu UH, Alfadda A, Sulimani RA, Al-Rubeaan KA, Al-Ruhaily AD, Fouda MA, Al-Maatouq MA, El-Desouki MI (2008). Graves’ disease in Saudi Arabia: a ten-year hospital study. J Pak Med Assoc.

[6] LaFranchi S. Clinical manifestations and diagnosis of Graves disease in children and adolescents. In: UptoDate. UptoDate; 2021.

